# Genomics Approaches for Crop Improvement against Abiotic Stress

**DOI:** 10.1155/2013/361921

**Published:** 2013-06-06

**Authors:** Bala Anı Akpınar, Stuart J. Lucas, Hikmet Budak

**Affiliations:** ^1^Faculty of Engineering and Natural Sciences, Sabanci University, Orhanlı, Tuzla, 34956 Istanbul, Turkey; ^2^Sabanci University Nanotechnology Research and Application Centre (SUNUM), Sabanci University, Orhanlı, Tuzla, 34956 Istanbul, Turkey

## Abstract

As sessile organisms, plants are inevitably exposed to one or a combination of stress factors every now and then throughout their growth and development. Stress responses vary considerably even in the same plant species; stress-susceptible genotypes are at one extreme, and stress-tolerant ones are at the other. Elucidation of the stress responses of crop plants is of extreme relevance, considering the central role of crops in food and biofuel production. Crop improvement has been a traditional issue to increase yields and enhance stress tolerance; however, crop improvement against abiotic stresses has been particularly compelling, given the complex nature of these stresses. As traditional strategies for crop improvement approach their limits, the era of genomics research has arisen with new and promising perspectives in breeding improved varieties against abiotic stresses.

## 1. Introduction

Abiotic stresses are the most significant causes of yield losses in plants, implicated to reduce yields by as much as 50% [1]. Among abiotic stresses, drought is the most prominent and widespread; consequently the drought stress response has been dissected into its components and extensively studied in order to understand tolerance mechanisms thoroughly [2]. To improve abiotic stress, particularly drought, tolerance of cereals is of extreme importance, as cereals, including wheat and barley, are the main constituents of the world food supply. However, many abiotic stresses are complex in nature, controlled by networks of genetic and environmental factors that hamper breeding strategies [3]. As traditional approaches for crop improvement reach their limits, agriculture has to adopt novel approaches to meet the demands of an ever-growing world population. 

**Figure 1:**
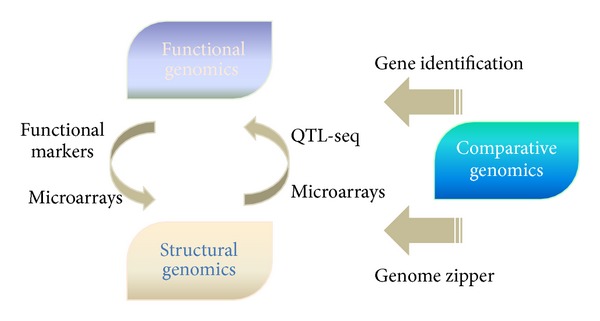
Functional, structural, and comparative genomics approaches are highly interrelated. For example, microarrays can be used either to anchor markers to genome maps or to analyze gene expression; functional markers indicate both phenotypes and genetic locations; QTL-seq utilizes a reference genome sequence to isolate QTLs based on phenotypic variation. As more structural genomics information becomes available, comparative genomics tools such as genome zippers can be used both to elucidate the structure of unsequenced genomes and as a shortcut to design targeted functional studies.

Recent technological advances and the aforementioned agricultural challenges have led to the emergence of high-throughput tools to explore and exploit plant genomes for crop improvement. These genomics-based approaches aim to decipher the entire genome, including genic and intergenic regions, to gain insights into plant molecular responses which will in turn provide specific strategies for crop improvement. In this paper, genomics approaches for crop improvement against abiotic stresses will be discussed under three generalized classes, functional, structural, and comparative genomics. However, it should be noted that genomics approaches are highly intermingled, in terms of both the methodologies and the outcome (Figure 1).

## 2. Functional Genomics

Genomics research is frequently realized by functional studies, which produce perhaps the most readily applicable information for crop improvement. Functional genomics techniques have long been adopted to unravel gene functions and the interactions between genes in regulatory networks, which can be exploited to generate improved varieties. Functional genomics approaches predominantly employ sequence or hybridization based methodologies which are discussed below. 

### 2.1. Sequencing-Based Approaches

One way to explore the expressed gene catalogue of a species is to analyze Expressed Sequence Tags (ESTs). ESTs are partial genic sequences that are generated by single-pass sequencing of cDNA clones [4]. Despite the concerns over the quality of ESTs as well as the representation of the parental cDNA [5], ESTs have been shown to identify corresponding genes unambiguously in a rapid and cost-effective fashion [4]; therefore, ESTs have been a major focus on functional studies. 

Large-scale EST sequencing has been one of the earliest strategies for gene discovery and genome annotation [5, 6]. Currently, over a million ESTs are deposited in the EST database at National Center for Biotechnological Information (NCBI) for important crops such as maize, soybean, wheat, and rice, along with several thousands of ESTs for other plants (http://www.ncbi.nlm.nih.gov/dbEST/). cDNA libraries from various tissues, developmental stages, or treatments generally serve as the sources for EST sequencing to reveal differentially expressed genes [7]. These approaches can successfully identify tissue or developmental stage-specific and treatment-responsive transcripts. However, such cDNA libraries may underrepresent rare transcripts or transcripts that are not expressed under certain conditions. In addition, ESTs are usually much shorter in length than the cDNAs from which they are obtained. Assembly of overlapping EST sequences into consensus contigs is likely to be more informative on the structure of the parental cDNA, which may reveal polymorphisms. However, assembly and interpretation must be handled cautiously, as paralogous genes may lead to misassemblies of sequences, particularly in polyploid species such as wheat [5]. EST sequencing is utilized extensively in the absence of whole genome sequences, particularly in crops with large and repetitive genomes, although the entire transcriptome is unlikely to be fully represented and resolved. Even so, EST sequencing is still a valid approach, and a recent study has demonstrated its potential in gene discovery via the comparison of different genotypes under control and stress conditions [8]. 

An alternative approach, Serial Analysis of Gene Expression (SAGE), has been developed to quantitate the abundance of thousands of transcripts simultaneously. In this approach, short sequence tags from transcripts are concatenated and sequenced, giving an absolute measure of gene expression [9, 10]. The ability of these short tags to identify genes unambiguously depends on the existence of comprehensive EST databases for the respective species [11]. Although SAGE is not widely applied in plants, there are a number of examples, including modifications to the original methodology, such as SuperSAGE and DeepSAGE [12–17]. The first report of SAGE in plants not only identified novel genes but also implied novel functions for known genes in rice seedlings [12]. SAGE has also been used to investigate stress-responsive genes [12, 15]. A similar tag-based approach, Massively Parallel Signature Sequencing (MPSS), where longer sequence tags are ligated to microbeads and sequenced in parallel, enables analysis of millions of transcripts simultaneously [18]. Due to longer tags and high-throughput analysis, MPSS is likely to identify genes with greater specificity and sensitivity. The ability of MPSS to capture rare transcripts is particularly beneficial in species that lack a whole genome sequence [19]. In plants, besides mRNA transcripts, MPSS has been employed in the expression studies of small RNAs [20, 21], which are increasingly implicated in abiotic stress responses [22]. Currently, plant MPSS databases (http://mpss.udel.edu/) contain publicly available MPSS expression data for a number of plant species, including important crops such as rice, maize, and soybean [23]. These MPSS data can be extracted, compiled, and compared with newly generated MPSS data for functional analysis of gene expression, as demonstrated by Jain et al. [24].

### 2.2. Hybridization-Based Approaches

In contrast to sequence-based approaches, array-based techniques utilize hybridization of the target DNA with cDNA or oligonucleotide probes attached to a surface to assess expression [25, 26]. These array-based methods are targeted; that is, prior knowledge of the transcript to be analyzed, either sequence or clone, is a prerequisite to design probes [27]. Extensive microarray expression data exists for *Arabidopsis thaliana* and rice [28–31], model species with fully sequenced genomes. In addition, microarray studies have been widely employed in crop species such as wheat [32], barley [33], and maize [34], as well as less emphasized but still industrially and agriculturally important plant species, such as cotton [35], cassava [36], and tomato [37] to unravel stress responses. 

Besides inherent limitations such as cross-hybridization and background noise, microarray studies investigating stress-responsive genes suffer from technical considerations that may limit their usefulness. Isolation of total RNA from complex tissues that are composed of different types of cells may obscure transcript changes occurring in cell types that are particularly relevant to the stress response. Subtle transcriptional changes may be diluted in the overall stress response of the whole tissue and, thus, remain unnoticed. Similarly, the choice of tissue or genotype that is sampled in a microarray study is closely related to the relevance of results. Reproductive tissues and stress-tolerant genotypes are most relevant in terms of agricultural gain and stress adaptation mechanisms, respectively [38]. In addition, laboratory-based stress treatments rarely represent field conditions, where multiple stresses usually act together. Interestingly, a comparison of microarray studies carried out using different water deficit stress conditions revealed only a small number of commonly regulated genes [39]. Abiotic stresses are generally complex in nature, eliciting intricate mechanisms of responses in plants. Consequently, slight differences in the experimental application of stress conditions may produce significant differences in stress responses. A further caveat when interpreting microarray studies is that many transcripts are known to undergo posttranscriptional and posttranslational modifications, which results in uncorrelated transcriptomic and proteomic data in some cases. 

For species with an available whole genome sequence, a successful expansion of array-based transcript profiling is whole genome tiling arrays [27]. Tiling arrays can identify novel transcriptional units on chromosomes and alternative splice sites and can map transcripts and methylation sites [40, 41]. Tiling arrays have already been applied in model species to investigate abiotic stress responses [42–44].

### 2.3. Expansions to Functional Genomics Approaches

Genome wide expression profiles are most useful in the detection of candidate genes for desired traits, such as stress tolerance. A fraction of functional studies then adopt inactivation or overexpression of such candidate genes for further characterization and utilization. Of these, Targeting Induced Local Lesions IN Genomes (TILLING) enables high-throughput analysis of large number of mutants [45]. TILLING is applicable to virtually all genes in all species where mutations can be induced and has been reported in several crop species, including hexaploid wheat [46]. TILLING mutants are reported in sorghum [47], maize [48], barley [49], soybean [50], rice [51], and other crops. Although TILLING populations are conventionally screened by phenotypic or genotypic variations, further use of certain TILLING mutants in elucidation of stress responses has been demonstrated. In such a study, TILLING mutants for a specific kinase were used to assess salt stress response in legume species [52]. 

Importantly, a modified strategy, called EcoTILLING, has been developed to identify natural polymorphisms, analogous to TILLING-assisted identification of induced mutations. Polymorphisms demonstrating natural variation in germplasms are valuable tools in genetic mapping. Furthermore, via the discovery of polymorphisms among individuals, EcoTILLING is able to implicate favorable haplotypes for further analyses, such as sequencing. Similar to TILLING, EcoTILLING is applicable to polyploid species, where it can be utilized to differentiate between alleles of homologous and paralogous genes [53]. In a recent study, EcoTILLING not only provided allelic variants of a number of genes involved in salt stress response but also emphasized the complex nature of salt stress; salt-tolerant genotypes were revealed to harbor different combinations of favorable alleles indicating the presence of multiple pathways conferring salt stress tolerance [54]. Transcription factors, diversifying stress responses, have also been targeted via EcoTILLING to examine natural rice variants exposed to drought stress [55]. 

The availability of comprehensive EST databases is central to the success of the above-mentioned approaches to identify genes accurately and unambiguously. Besides their utility in genome annotation and expression profiling, ESTs also provide a source of sequences for designing “functional markers.” Functional markers refer to polymorphic sites on genes that are attributed to phenotypic variation of traits among individuals of a species. Functional marker design requires the knowledge of the allelic sequences of functionally characterized genes [56]. In contrast to random DNA markers, functional markers are completely linked to the trait of interest; hence, these markers are also called “perfect markers.” The use of random DNA markers in breeding studies necessitates validation and revalidation of linkage between the marker and the trait over generations, since genetic recombination may break the linkage [56, 57]. In addition, functional markers may explore natural variation and biodiversity better, particularly compared to random DNA markers with absence/presence polymorphisms, where allelic variations of a trait exceed that of the linked DNA marker. In the case of such random DNA markers, the locus tested during genotyping will only exhibit biallelic variation, whereas the linked gene may actually have more variants [56]. The importance of functional markers has been highlighted in stress tolerance studies as well [58, 59].

## 3. Structural Genomics

While functional genomics focus on the functions of genes and gene networks, structural genomics focus on the physical structure of the genome, aiming to identify, locate, and order genomic features along chromosomes. Together, structural genomics and functional genomics can characterize a genome to its full extent. 

### 3.1. Genome Sequencing and Mapping

In the last decade, advances in DNA sequencing technologies have enabled the generation of a wealth of sequence information including whole genome sequences. Next-generation sequencing (NGS) platforms such as Roche 454 GS FLX Titanium (http://www.454.com/) or Illumina Solexa Genome Analyzer (http://www.illumina.com/) can carry out high capacity sequencing at reduced costs and increased rates compared to conventional Sanger sequencing [60]. These advances have paved the way for the exploitation of plant genomics studies for breeding improved varieties. Through NGS technologies, sequencing and resequencing of even large genomes have become feasible. Accordingly, reference or draft genome sequences for a number of species, including the model species *Arabidopsis thaliana* and *Brachypodium distachyon*, along with important crop species such as rice, sorghum, soybean, and maize, have been published [61]. Whole genome sequences provide remarkably detailed information on genomic features including coding and noncoding genes, regulatory sequences, repetitive elements, and GC content which can be exploited in functional studies such as microarray or tiling arrays [41]. A high-quality reference genome sequence is considered pivotal to crop improvement via molecular breeding, particularly for complex traits. Despite their usefulness, producing such reference genomes requires a major investment of resources, and currently they are only available for species with relatively small genomes of low repetitive content [61].

*Triticeae* genomics, including that of the staple crops barley and wheat, has lagged behind recent advances primarily due to their large and complex genomes (~5 Gb for barley and ~17 Gb for wheat) [62]. As pointed out by Morrell et al. [61], 25x coverage sequencing of *Drosophila* is equivalent to approximately 1x coverage of wheat genome in terms of sequence read counts, demonstrating the challenging genome size of wheat. The high content of repetitive elements is another major challenge, causing ambiguities in sequence assembly. In polyploid species such as wheat, the sequence assembly problem is further exacerbated due to the presence of homoeologous genomes and paralogous loci [61]. For such genomes, construction of a reference sequence has been considered unattainable until recently. 

Over the last few years, advances in chromosome sorting technologies have enabled construction of chromosome-specific Bacterial Artificial Chromosome (BAC) libraries to tackle the challenges of complex genomes. Physical mapping of the 1 Gb chromosome 3B of hexaploid wheat has proven the feasibility of a chromosome-by-chromosome approach to explore and exploit complex genomes [63]. Physical maps not only compile genetic mapping data into physical contigs but also serve as scaffolds for sequence assembly into a reference genome. The physical mapping and reference genome sequencing of wheat and barley are ongoing with combined efforts from a number of consortia [62].

In the absence of reference genome sequences, whole genome or BAC-end shotgun sequences provide valuable insights into genome structure and evolution [64–69]. Intriguingly, whole genome shotgun sequences have also been proposed for Quantitative Trait Loci (QTL) detection via a very recently developed methodology named QTL-seq. In this method, extremes of a population exhibiting a normal distribution with respect to a trait of interest are bulked, sequenced, and compared to detect putative QTLs [70]. 

### 3.2. Molecular Markers

Genomics applications involving molecular markers are largely dominated by Single Nucleotide Polymorphisms (SNPs) [71] as reflected in the predominance of software related to SNP discovery [60]. The high abundance of SNPs in genomes is particularly beneficial for their use in genomics. SNPs are readily identified by genome or transcript resequencing and by comparison of different genotypes in species where reference genome sequences or extensive transcript databases are available. Transcriptome resequencing not only avoids repetitive sequences of complex genome but also identifies SNPs within transcripts that may serve as functional markers [72]. However, due to low-quality sequences obtained by most NGS platforms, over-sampling may be required to differentiate SNPs from sequencing errors [71]. In addition, the presence of homoeologous and paralogous loci must be taken into account in SNP identification in polyploid species [72]. Despite the challenges of SNP discovery on the repetitive portion of genomes, efforts are underway to improve SNP identification even in gene-poor regions [73]. In fact, these regions are of functional importance as well; for example, an important vernalization gene *Vrn-D4* has recently been mapped to the centromeric region of chromosome 5D of hexaploid wheat [62, 74].

A recently developed molecular marker type, Insertion Site-Based Polymorphisms (ISBPs), utilizes the insertional polymorphisms observed in the repeat junctions of complex genomes [75]. ISBP markers are readily designed from low coverage shotgun sequences, such as BAC-end sequences [64, 69]. Typically, 50–60% of ISBP markers tested are specific for the locus from which they were designed, and in one study which these ~70% contained SNPs in at least some members of a panel of 14 wheat genotypes [75]. This approach may break the ground for genome saturation particularly for crops with highly repetitive genomes that are impractical to exploit otherwise.

### 3.3. Applications of Structural Genomics in Crop Improvement

A major impact of NGS-mediated shotgun sequences has been their substantial contribution to the development of molecular markers. These markers indicate diagnostic polymorphisms at the DNA sequence level, and in contrast to morphological markers which once had been the focus of traditional breeding studies, they are not affected by the environment [76]. In general terms, Marker-Assisted Selection (MAS) refers to the utilization of molecular markers in breeding improved varieties with respect to desired traits, such pathogen resistance, abiotic stress tolerance, or high yield [77]. Through MAS, phenotype can be predicted from genotype [71]. For efficient and accurate MAS, the trait of interest should be tightly linked to a molecular marker [78] or more preferably flanked by two close markers. Recombination between both flanking markers and the trait is less likely to occur compared to a single marker, due to the low frequency of double crossovers. In both cases, a genetic distance of less than 5 cM for each marker from the trait is crucial to the success of MAS [77]. 

Additionally, for efficient MAS, markers should be highly polymorphic in the germplasm used for breeding. MAS can make use of molecular markers at multiple levels. Plant breeding depends on genetic diversity to improve crops [57]. Molecular markers may aid in the exploration of the variation among the germplasm to select the best candidate parental lines. Similarly, molecular markers may identify heterotic groups or ensure genomic purity of cultivars to achieve heterosis. In addition, molecular markers also assist in backcrossing. Plant breeding conventionally involves several backcrossing steps to enable transfer of one or a few traits to an elite cultivar while retaining most of the recurrent genomes. In general, at least six rounds of backcrossing are required to achieve the desired homozygosity, particularly for the selection of traits with low heritability. In contrast, MAS can greatly accelerate this process by utilizing both the flanking markers linked to the trait for selecting the trait and a set of unlinked markers for tracking the recurrent genome. Flanking markers and selection for recombination also reduces “linkage drag,” which is the reduction in crop performance due to the cotransfer of undesirable traits that are located in the vicinity of the trait of interest [77]. Typically, a conventional QTL analysis can provide a resolution of approximately 15 cM intervals which may contain hundreds of genes [79]. The availability of a saturated map can potentially reduce this interval to less than 1 cM by backcrossing [78]. Furthermore, MAS enables early selection of traits that are labor and/or cost-intensive to score phenotypically, that are under complex genetic control, or that are manifested late in development. In cases where genotyping by MAS is affordable, this dramatically reduces the number of the plants to be screened in further steps [77, 78]. 

The major drawbacks of MAS in breeding are high costs of implementation, typically requiring specialized equipment, and the risk of recombination between the marker and the trait that reduces the reliability of MAS to predict phenotype via genotype. The high cost of MAS is particularly relevant in cases where an effective phenotyping method is already established through conventional breeding. Additionally, MAS usually requires the validation of QTLs when applied in different genetic backgrounds. Functional markers, however, may overcome the issue of QTL validation [78]. Despite its drawbacks, MAS has been successfully utilized to improve crops for abiotic stress tolerance, including drought [80], salinity [81], and waterlogging [82] given that the genetic element responsible for the high tolerance is accurately defined and delineated. 

Another use of molecular markers is Map-Based Cloning (MBC) where the gene or a QTL linked to a desired trait is isolated via a “mini” physical map. Such a local physical map flanking the gene must be saturated with molecular markers for efficient MBC [6]. Prior to the construction of high-density physical maps, MBC approaches were inefficient, particularly due to the difficulty of finding unique probes in repetitive sequences for chromosome walking. Importantly, repeat contents of barley and wheat genomes, two staple crops, are estimated to exceed 80% of the whole genome [83, 84], potentiating the utility of physical maps. Accordingly, the physical map of chromosome 3B provided sufficient data to enable fine mapping of 16 genes and QTLs in chromosome 3B, none of which had been previously cloned [64]. 

## 4. Comparative Genomics

**Figure 2:**
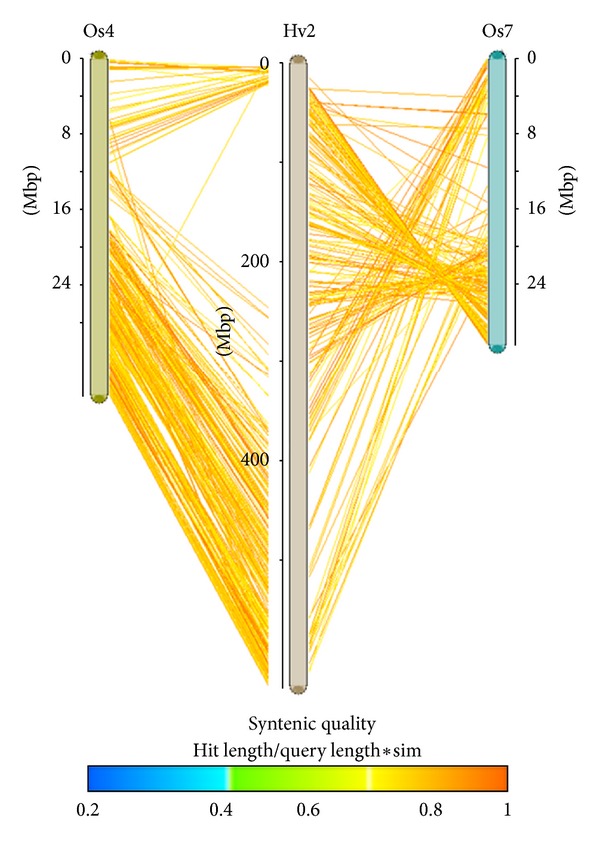
Example of colinearity between grass genomes. Analysis of conserved gene sequences between barley (*H. vulgare*) and rice (*O. sativa*) shows that many genes are found in colinear (syntenic) order. In this example, chromosome 2 of barley (Hv2, centre) is compared with chromosomes 4 and 7 of rice (Os4 and Os7). Each colored line represents a gene conserved between the two chromosomes, with the color indicating the strength of the syntenic relationship. It is clear that many genes from both ends of Hv2 are colinear with the ends of Os4, while the centre of the chromosome is largely colinear with Os7, but in the reverse order. Image was generated using the CrowsNest Comparative Map Viewer at MIPS (http://mips.helmholtz-muenchen.de/plant/genomes.jsp).

For species with largely unexplored genomes, comparative genomics is a promising tool to gain information by utilizing the conservation between closely related plant species. In fact, plant genomes share extensive similarities even between distantly related species (Figure 2, [85]). Among the plant kingdom, grasses have been the focus of comparative genomics analyses due to their high agronomic importance. The extent of genome conservation first became evident by comparative genome mapping studies, which suggested a colinear order of genes and markers shared by genomes of different species. It is noteworthy that plant genomes differ by several orders of magnitude in size; yet these differences generally correspond to intergenic regions. Although detailed analyses have revealed notable rearrangements such as inversions, deletions, and translocations at the molecular level, large-scale colinearity across grass genomes has been exploited for gene discovery and isolation [86–88].

Comparative genomics has contributed significantly to the emergence of the “genome zipper” concept, which enables the determination of a virtual gene order in a partially sequenced genome. Genome zippers compare the fully sequenced and annotated genomes of *Brachypodium*, sorghum, and rice with various sources of data from less well-studied species, such as genomic survey sequences and genetically mapped markers, to predict the gene order and organization in these species [65, 89]. These genome zippers indicate evolutionary relationships and medium-scale rearrangements, and for the *Triticeae* provide the closest approximation to a reference genome sequence currently available [65]. However, its reliance on synteny means that recently evolved genes and small-scale rearrangements cannot be explored by this approach.

In addition to syntenic genes that are found in colinear blocks of conserved genes, nonsyntenic genes that are found outside their syntenic location in other genomes also provide valuable insight into genome evolution and speciation. Intriguingly, a recent study focused on the nonconserved portion of wheat and barley genome that suggested novel mechanisms, besides transposable element-driven expansion, have driven the evolution and size of these genomes. Many of these non-syntenic genes exhibited pseudogene characteristics, which may have implications for gene content estimate of these large genomes based on survey sequences [90].

Despite the utility of comparative genomics and genome zippers, it is evident that species-specific genomic features can only be accessed through a fully annotated reference genome sequences. Homoeologous genes with different orthologous relationships are examples of such species-specific features [91]. Species-specific rearrangements are also implicated in the formation of gene islands, containing mainly non-syntenic genes, in large crop genomes [92]. Through comparative genomics, Mayer et al. [65] concluded that genomic models can represent the barley genome to a limited extent. Thus, it can be argued that for maximal exploitation of crop genomes, such as wheat and barley, the construction of reference genome sequences scaffolded by highly-saturated physical and genetic maps is indispensable. Accordingly, efforts to accomplish this goal are currently underway (International Wheat Genome Sequencing Consortium for wheat; International Barley Sequencing Consortium for barley).
